# Toxoplasmosis in a bar-shouldered dove (*Geopelia humeralis*) from the Zoo of Clères, France

**DOI:** 10.1051/parasite/2014062

**Published:** 2014-11-20

**Authors:** Jacques Rigoulet, Alain Hennache, Pierre Lagourette, Catherine George, Loïc Longeart, Jean-Loïc Le Net, Jitender P. Dubey

**Affiliations:** 1 Muséum National d’Histoire Naturelle, Département des Jardins Botaniques et Zoologiques 57 rue Cuvier 75005 Paris France; 2 Laboratoire Anatomo-Pathologique Vétérinaire BP 303 37403 Amboise France; 3 United States Department of Agriculture, Agricultural Research Service, Beltsville Agricultural Research Center, Animal Parasitic Diseases Laboratory Beltsville Maryland 20705-2350 USA

**Keywords:** *Toxoplasma gondii*, toxoplasmosis, bar-shouldered dove, *Geopelia humeralis*, France

## Abstract

Toxoplasmosis causes mortality in several avian species, especially passerine birds. Toxoplasmosis was diagnosed in a bar-shouldered dove (*Geopelia humeralis*) found dead at the zoo of Clères (France). The bird had necrotizing pneumonia and nephritis with intralesional tachyzoites of *Toxoplasma gondii.* The diagnosis was confirmed by immunostaining with polyclonal rabbit *T. gondii* antibodies and by transmission electron microscopy. To our knowledge, the bar-shouldered dove is a new host record for *T. gondii*.

## Abstract (fr)

La toxoplasmose est une cause de mortalité chez plusieurs espèces d’oiseaux, en particulier les passereaux. La toxoplasmose a été diagnostiquée chez une Géopélie à nuque rousse (*Geopelia humeralis*) retrouvée morte au zoo de Clères (France). L’oiseau avait une pneumonie nécrosante et une néphrite avec tachyzoïtes de *Toxoplasma gondii* intralésionnels. Le diagnostic a été confirmé par marquage immunologique avec des anticorps polyclonaux de lapin anti-*T. gondii* et par microscopie électronique à transmission. À notre connaissance, la Géopélie à nuque rousse est un nouvel hôte pour *T. gondii*.

## Introduction

*Toxoplasma gondii* can cause mortality in many species of mammals and birds [1]. Worldwide reports of toxoplasmosis in all avian species have recently been summarized [1, 2]. The passerine birds are especially susceptible to clinical toxoplasmosis [4, 8]. Pigeons can die of natural *T. gondii* infection, depending on the breed and age of the host [2, 3]. We report fatal toxoplasmosis in a bar-shouldered dove (*Geopelia humeralis*), which is to our knowledge the first report of *T. gondii* infection in this host.

## History

Since 1919, the zoological park of Clères (France) owns an important collection of captive birds, including Columbidae. Among Columbidae, *Geopelia humeralis* is an endemic species of East and North Australia, which breeds well in captivity. *Geopelia humeralis* is monogamic; the female lays two eggs, twice a year. The incubation period is 13–14 days; sexual maturity is reached at about one year; lifespan is 18 years, but reproduction stops at 12 years. The first couple of male and female *G. humeralis* was brought to Clères from the Taronga Zoo, Sydney, Australia at the end of the 1960s. It began to reproduce in 1970, and in 1996, the zoological park obtained the eighth generation of these doves. All these bar-shouldered doves are exhibited for the public, in large aviaries.

Toxoplasmosis was diagnosed the first time in a 6-year-old male *G. humeralis* (bird A); the bird was born in August 1989 and died at the end of December 1995. A female (bird B) that lived in the same aviary died one year later. A retrospective search of the zoo records revealed that two other (C and D) 7-year-old *G. humeralis* died in May–June 1995. Lack of appetite, asthenia, decline in health, and quick death were the recorded clinical signs. The present report is based on findings observed in bird A. Samples were not taken from others birds (B, C, and D).

## Materials and methods

A full necropsy was performed. Hemorrhage and pale areas were seen in lung. Portions of lung were fixed in 10% buffered formalin processed routinely in an automatic tissue processor, embedded in paraffin, sectioned at 5 μm, and stained with hematoxylin and eosin (H&E). Paraffin blocks of lung were sent to the Animal Parasitic Diseases Laboratory, US Department of Agriculture, where deparaffinized sections were stained with anti-*T. gondii* and anti-*Neospora caninum* antibodies following methods described previously [5]. For electron microscopic examination, 1-mm^3^ formalin-fixed samples were immersed in 2.5% glutaraldehyde in 0.1 M phosphate buffer, post-fixed in 2% osmium tetroxide, and embedded in epoxy resin. Semithin sections were stained with toluidine blue, and ultrathin sections were stained with uranyl acetate and lead citrate and examined with a Zeiss EM109 electron microscope.

## Results and discussion

**Figure 1.**
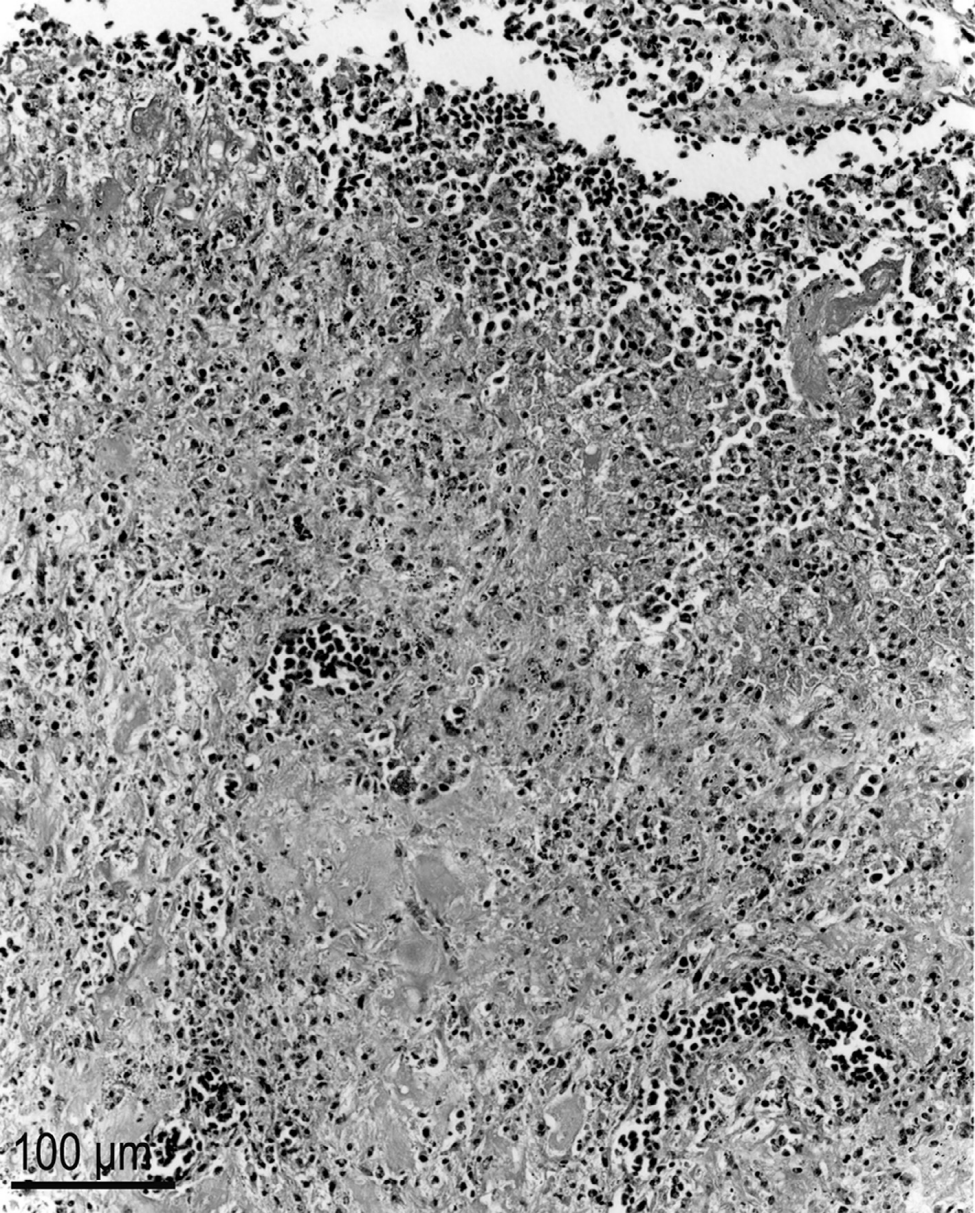
*Toxoplasma gondii* in a bar-shouldered dove, lung. Note the necrotic process. There are numerous intralesional tachyzoites but non-visible at this magnification. H&E stain.

**Figure 2.**
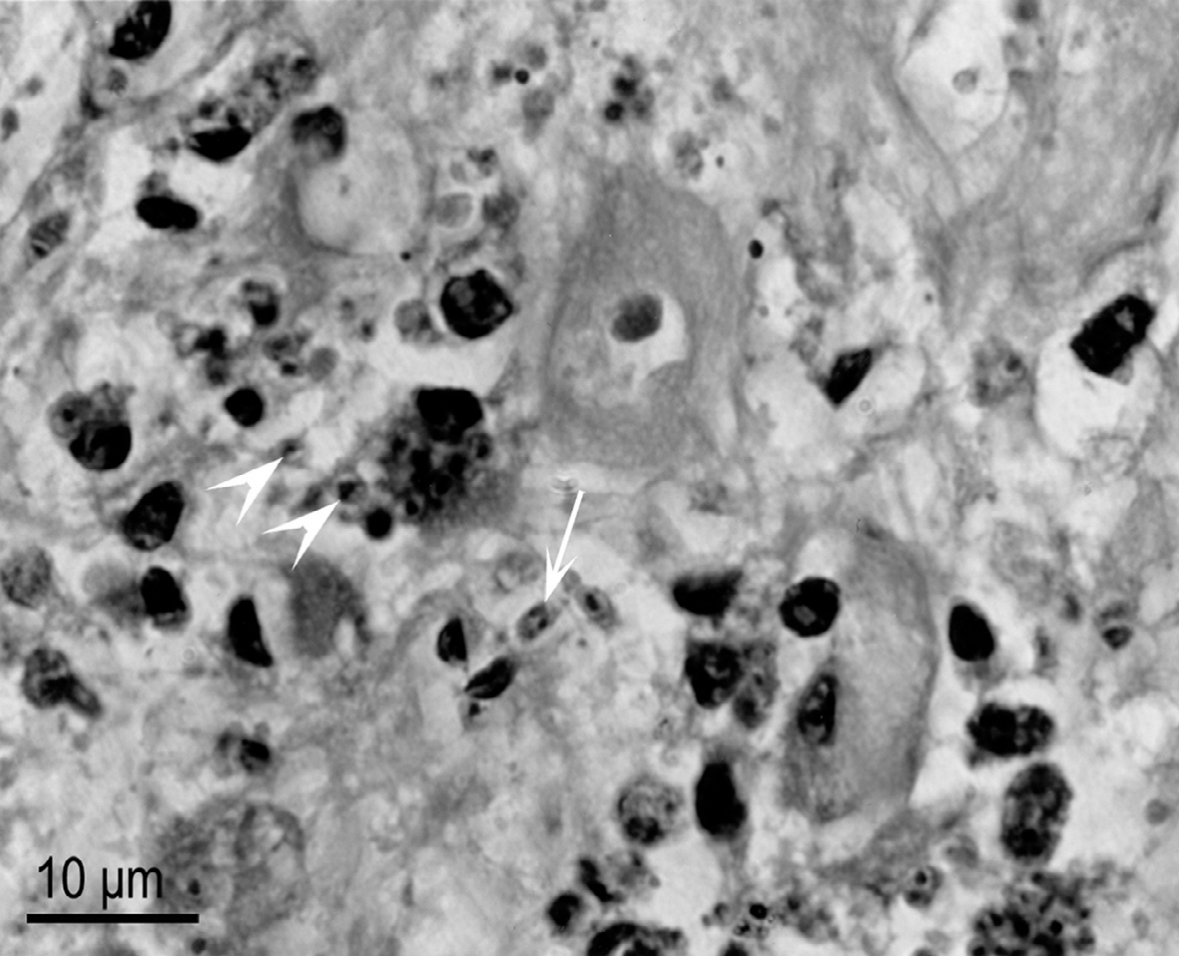
*Toxoplasma gondii* in a bar-shouldered dove, lung. Note a tachyzoite with dividing nucleus (arrow) and individual tachyzoites (arrowheads). H&E stain.

Necrotizing pneumonia associated with numerous protozoa was observed microscopically (Fig. 1). In H&E sections, the protozoal tachyzoites had a central nucleus and a pale staining cytoplasm; they were 2–3 μm in diameter and most of them showed evidence of degeneration (Fig. 2). The protozoa reacted strongly to *T. gondii* and not to anti-*N. caninum* antibodies.

**Figure 3.**
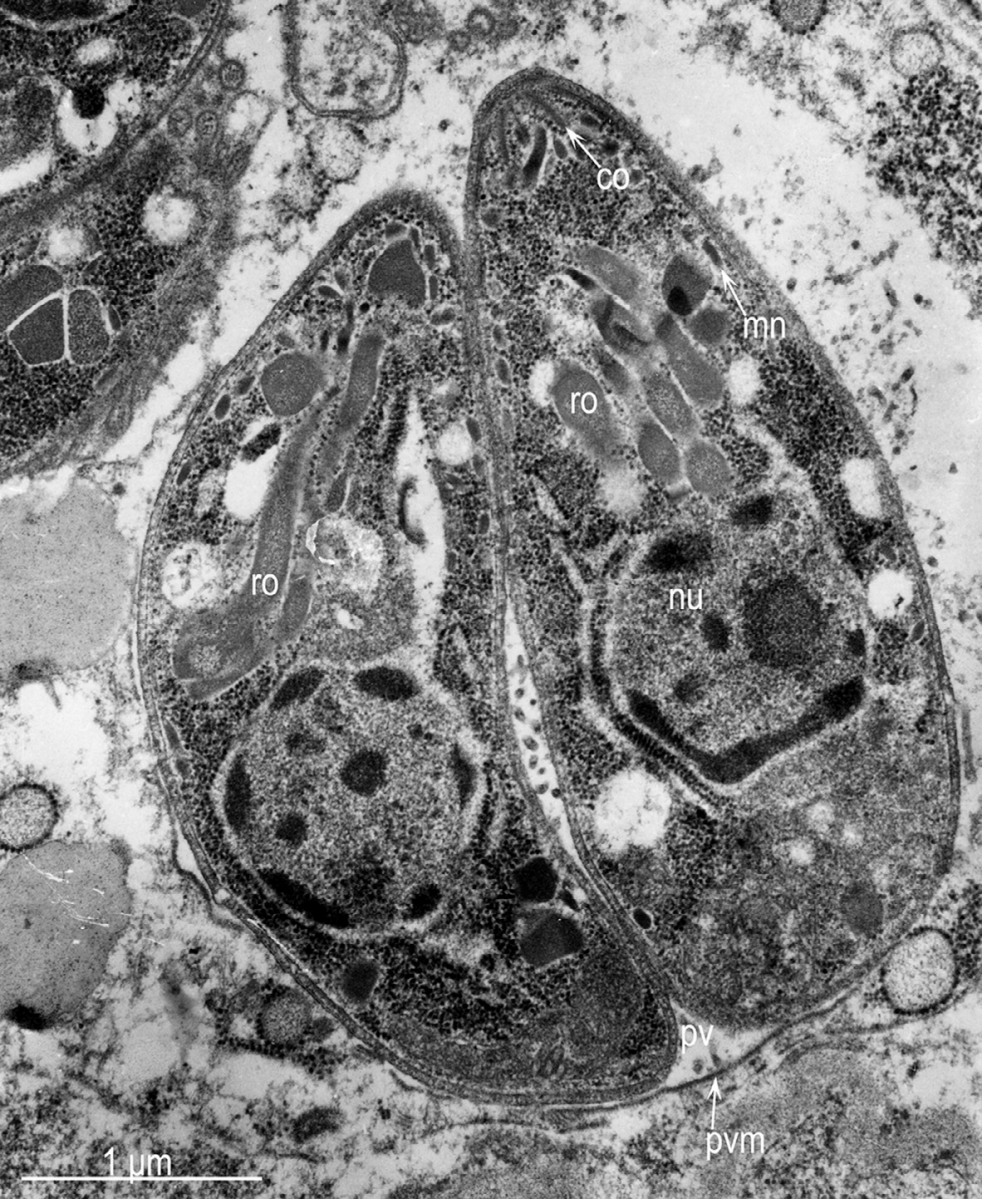
*Toxoplasma gondii* in a bar-shouldered dove, lung. Two tachyzoites enclosed in a parasitophorous vacuolar membrane (pvm). Note conoid (co), micronemes (mn), rhoptries (ro) with honey-combed contents, and a nucleus (nu) in each tachyzoite. The parasitophorous vacuole has membranous tubules. Transmission electron microscopy.

Ultrastructurally, the protozoa were located in parasitophorous vacuoles in the host cell cytoplasm. Organisms divided in two by endodyogeny (Fig. 3). Organelles typical of *T. gondii* tachyzoites were seen, including micronemes and rhoptries with labyrinthine contents.

The present case was diagnosed as toxoplasmosis based on ultrastructure and immunoreactivity to *T. gondii*. Diagnosis of avian toxoplasmosis is often difficult. Species of two related protozoans, *Atoxoplasma* and *Sarcocystis*, should be considered in the differential diagnosis of avian toxoplasmosis [1, 6, 7]. *Atoxoplasma* spp. are considered common parasites of passerine birds and have a fecal-oral cycle with extra-intestinal stages in visceral tissues of birds, especially the liver and spleen [1]. Proliferative stages (merozoites) of *Atoxoplasma* sp. are smaller than *T. gondii* tachyzoites. Ultrastructurally, *Atoxoplasma* merozoites divide by schizogony, have small vestigeal rhoptries, and small numbers of micronemes [1]. The parasite in the present case divided by endodyogeny and had long rhoptries.

*Sarcocystis* spp. (*S. falcatula* or *S. falcatula*-like) can cause generalized disease in birds, especially in passerines and psittacines [4, 7]. Pneumonia is the predominant lesion of acute *S. falcatula* infection and disease is associated with intravascular development of *S. falcatula* schizonts [7]. Additionally, *S. falcatula* infections are confined to the geographical distribution (Americas) of the definitive host, opossum (*Didelphis* spp.). Recently, another species of *Sarcocystis, S. calchasi* was identified to cause fatality in racing pigeons in Europe and the Americas [6, 9]. However, *Sarcocystis* schizonts divide by endopolygeny (multiple nuclear lobulation), and merozoites lack rhoptries [1]. Additionally, the polyclonal antibodies used in the present study do not cross react with *Sarcocystis* and *Atoxoplasma* (Dubey, own observations).

These four bar-shouldered doves were probably exposed to a strain of *Toxoplasma gondii* virulent for birds in 1995.
